# Complex regulation of the sirtuin-dependent reversible lysine acetylation system of **Salmonella enterica**

**DOI:** 10.15698/mic2015.11.239

**Published:** 2015-10-12

**Authors:** Kristy L. Hentchel, Jorge C. Escalante-Semerena

**Affiliations:** 1 Department of Microbiology, University of Georgia, Athens, GA 30602, USA.; 2 Current address: Department of Biochemistry & Molecular Biology, University of Chicago, Chicago, IL 60637, USA.

**Keywords:** sirtuin deacetylase (CobB), protein acetyltransferase (Pat), reversible lysine acetylation (RLA), transcriptional regulation, IolR, myo-inositol utilization, Salmonella

## Abstract

The extensive involvement of the reversible lysine acylation (RLA) system in metabolism has attracted the attention of investigators interested in understanding the fundamentals of prokaryotic and eukaryotic cell function. Research in this area of cell physiology is diverse, ranging, among others, from probing the molecular bases of human diseases, to optimizing engineered metabolic pathways for biotechnological applications, to advancing our understanding of fundamental cellular processes. A gap of knowledge exists in our understanding of the regulatory circuitry that integrates the expression of genes encoding modifiers (i.e., acyltransferases) and demodifiers (i.e., deacylases) with the expression of genes encoding known targets of the system. Here we discuss the implications of recently reported work performed in the enteropathogen *Salmonella enterica* (*mBio *(2015) 6(4):e00891-15), which provided the first insights into the integration of the transcriptional regulation of genes encoding the RLA system with the *acs *gene encoding the central metabolic enzyme acetyl-CoA synthetase (Acs).

Lysine acetylation has been known for over 50 years to play an important role in histone-mediated control of eukaryotic gene expression, a process known to require the function of silent information regulatory (Sir) proteins. In the late 1990s our group showed that a homologue of the yeast Sir2 protein (aka, sirtuin) found in *Salmonella enterica *was an enzyme that used a pyridine nucleotide as co-substrate and functioned in the context of central metabolism. That finding unveiled, for the first time, a link between sirtuin function and metabolism. Subsequent studies by our research group, and others, firmly established the role of sirtuins as protein deacetylases. Our group also identified a homologue of the yeast Gcn5-type histone *N*-acetyltransferase (GNAT) enzyme in *S. enterica *(known as Pat, for protein acetyltransferase), which, in concert with the CobB sirtuin, comprise the reversible lysine acetylation (RLA) system of this bacterium. Collectively, the *S. enterica *Pat/CobB-dependent modulation of the activity of acetyl-CoA synthetase (Acs) became the paradigm for the role of reversible lysine acetylation in metabolism of cells from all domains of life, including humans.

In *Escherichia coli *(a close relative of *S. enterica*), *acs* expression is tightly controlled by several transcriptional regulators*. *In addition to this intricate interplay of transcriptional control the Pat acetyltransferase and CobB sirtuin modulate Acs activity, and do so by reversibly acetylating a lysine residue in the active site of Acs (Fig. 1A). Insights into the physiological need for posttranslational control were obtained when we demonstrated that Acs activity was dysregulated in the absence of Pat leading to an energy imbalance that led to growth arrest. Collectively, the evidence supports the conclusion that control of Acs activity by both transcriptional regulation and posttranslational modification is needed for balanced growth.

**Figure 1:**
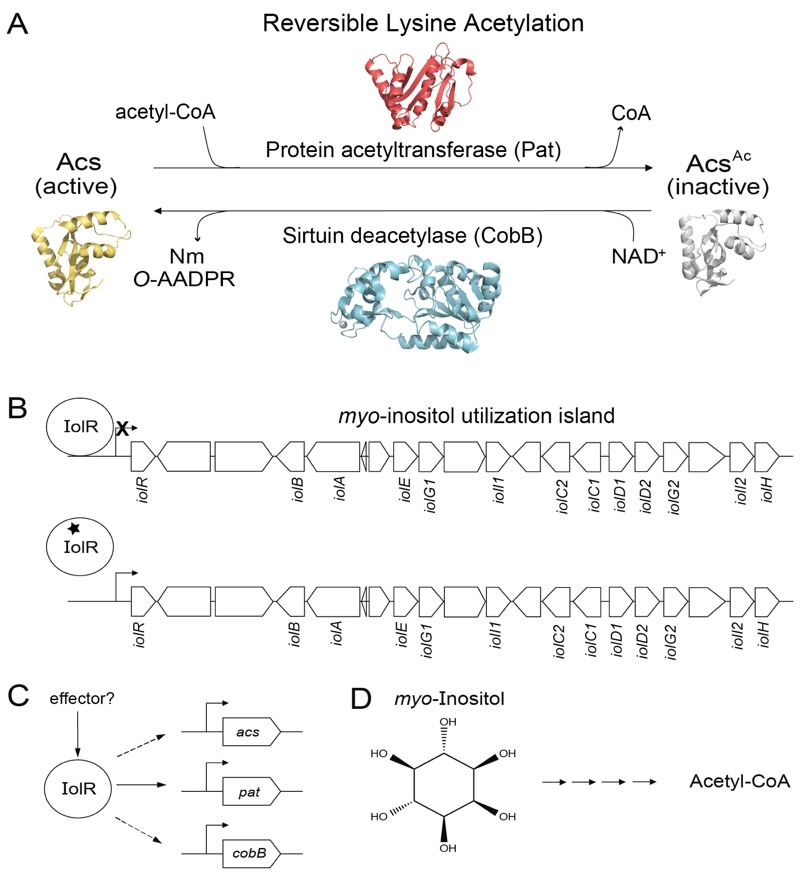
FIGURE 1: IolR activates expression of the reversible lysine acetylation (RLA) system in *S. enterica* and integrates it with that of its substrate, the acetyl-CoA synthetase, Acs. **(A)** In *S. enterica*, the activity of the AMP-forming acetyl-CoA synthetase (Acs) is post-translationally modified by the protein acetyltransferase, Pat. This modification is reversible by the activity of the NAD^+^-consuming class III sirtuin deacetylase, CobB, which releases the products *O*-acetyl-ADP-ribose (*O*-AADPR) and nicotinamide (Nm). **(B)** IolR binds to and represses transcription of the *myo*-inositol utilization island when *myo*-inositol is not available. Repression is relieved through binding of 2-deoxy-5-keto-D-gluconic acid 6-phosphate (**black star**), a product of *myo*-inositol metabolism, to IolR. **(C)** IolR is responsible for activation of expression of *pat*, *acs*, and *cobB*. The identity of a potential effector of this process is unknown. **(D)** Structure of *myo*-inositol, whose degradation leads to the generation of acetyl-CoA.

Until recently there was little information available regarding the transcriptional control of *cobB* and *pat*, and we did not know whether their expression was integrated in any way nor did we know which regulatory proteins were involved. To address these questions we initiated studies that provided the first insights into the regulatory circuitry that integrates *pat, cobB* and *acs *expression in *S. enterica*. Our initial findings were recently reported in *mBio *(2015) 6(4):e00891-15.

The following paragraph summarizes the findings reported in the above-mentioned paper. Briefly, *in vivo* growth experiments revealed that a ∆*iolR* strain had a substantial growth defect when grown with 10 mM acetate as the sole carbon source, a condition in which RLA control of Acs function is crucial. Collectively, the data supported the conclusion that the observed phenotype was due to an imbalance of the active (non-acetylated)/inactive (acetylated) Acs ratio caused by changes in *pat* and *cobB* expression in the absence of IolR. The growth defect of the Δ*iolR *strain was corrected by increasing the levels of Pat, CobB, or Acs activity. These results suggested that an altered Pat/CobB ratio was responsible for the growth defect of the Δ*iolR *mutant. The finding that the activity of Acs was decreased ~25% in a ∆*iolR* strain relative to the wild-type strain further substantiated this hypothesis. In summary, our work placed the RLA system and its *bona fide* target, Acs, under the transcriptional control of IolR.

We were surprised to find out that IolR, the repressor of the *myo-*inositol catabolic (*iol*) genes in *S. enterica*, plays a role in the expression of *pat, cobB*, and *acs. *This result was unexpected because IolR was previously reported to be a repressor, and there was no obvious explanation as to why IolR would be required to activate *pat, cobB *or *acs* expression. The above-mentioned work has raised interesting questions, such as: i) how does IolR activate *pat* expression? ii) is an effector (i.e., signal molecule) needed to modulate the affinity of IolR for the *pat *promoter? iii) what physiological conditions trigger *myo-*inositol-independent, IolR-dependent control of *pat, cobB *and* acs* expression? and iv) if the effect of IolR on *acs *and *cobB *expression is indirect, are there additional regulatory proteins and effectors involved in the process, and if so, what is their identity?

In thinking about how IolR may affect *cobB *and *acs *expression we are considering two possible scenarios. The first scenario is straightforward, that is, IolR directly binds to the *cobB *and *acs *promoters. At present, the identity of a IolR consensus DNA-binding site has not been resolved, and results from bioinformatics analyses have not identified any putative IolR binding sites within the *acs* and *cobB* promoters. The second scenario has a broader scope and implies the existence of additional, indirect layers of regulation. Regardless of the mechanism, we speculate that a metabolite, other than the one derived from *myo-*inositol metabolism (i.e., 2-deoxy-5-keto-D-gluconic acid 6-phosphate), is needed for IolR recognition of the *pat *promoter, and probably for the expression of *cobB *and *acs. *

One big picture question that must be addressed is why is the utilization of this cyclic polyalcohol as a sole carbon source interconnected with reversible lysine acetylation control of Acs. In addition, since *iolR* is located within the pathogenicity island 1 (SPI1) of *S. enterica*, it is possible that RLA control of Acs is important to the pathogenic lifestyle of this bacterium (Fig. 1B). Inositol and inositol phosphates are very abundant in plants, soil and many foods including fruits, grains, seeds, and beans. Hence, it is plausible that bacteria are exposed to *myo*-inositol and its various forms in the human gut, as well as the soil.

Because *myo-*inositol catabolism is a metabolic capability present in Gammaproteobacteria, Alphaproteobacteria, and some Gram-positive bacteria, it is likely that the connection between RLA control of Acs and *myo-*inositol is a widespread regulatory feature among prokaryotes, and if so, why? We consider the availability of genetic systems to be critical for advancing this field of research. The powerful genetic system of *S. enterica *makes this bacterium an ideal model organism for the investigation of this complex problem. Genetic approaches will likely identify the different components of the regulatory circuitry, and shed light into how it works. We see a potential pattern of carbon regulators playing a role in the modulation of expression of genes encoding RLA enzymes and its target Acs. Notably, the *S. enterica *catabolite repressor protein (CRP) activates expression *pat *and *acs,* but not *cobB*. These regulatory differences offer opportunities to search for mutant strains displaying changes in the original phenotypes. Biochemical and biophysical approaches will be needed to probe for the existence of putative effectors that may be required to direct IolR to the *pat *promoter (Fig. 1C), and to elucidate the chemical nature of it.

An additional area of interest is the connection between *myo-*inositol catabolism and C2 metabolism, since the breakdown of this polyalcohol yields acetyl-CoA (Fig. 1D). We are considering the possibility that when *S. enterica *(and maybe other prokaryotes) is growing on *myo-*inositol as the sole source of carbon and energy the intracellular level of acetyl-CoA rises to a point that compromises the level of free CoA needed for other purposes, thus triggering CoA biosynthesis and recycling. The latter can be accomplished in several ways: i) by excreting acetate via the phosphotransacetylase/acetate kinase (Pta/Ack) system with the benefit of generating energy via substrate level phosphorylation; ii) by using thioesterases to release CoA and acetate; or iii) by somehow accelerating the consumption of acetyl-CoA for anabolic and regulatory purposes. The latter point presents us with the opportunity to investigate whether the synthesis of specific acetyltransferases is increased during growth on *myo-*inositol, and what their targets may be.

In closing, reversible lysine acetylation is a regulatory mechanism present in all domains of life that helps to modulate the function of proteins involved in diverse cellular processes. The study reviewed herein offered the first insights into the intricacies of the system responsible for controlling RLA at the transcriptional level in the enteropathogenic bacterium *S. enterica* by beginning to uncover the regulatory circuitry that integrates the expression of genes encoding the RLA system with a central metabolic enzyme involved in C2 metabolism.

